# Photophysics of Self-Assembled PHE-PHE/PVK Systems Studied by FLIM

**DOI:** 10.1007/s10895-026-04860-y

**Published:** 2026-07-06

**Authors:** Tatiana Ertner, Diericon Cordeiro

**Affiliations:** Chemistry Institute, Federal University of Goiás, Goiania, 74690900 Brazil

**Keywords:** FLIM, Phe-Phe nanotube, Phe-Phe vesicle, Self-assembling, PVK, Fluorescence lifetime, FRET

## Abstract

Diphenylalanine (Phe-Phe) dipeptide can self-assemble into distinct structures, depending on the preparation conditions, which also influence its photophysical behavior. Therefore, to better understand the energy transfer processes to which those structures are subject and aim an application, it is crucial to find the best techniques that are able to explore the reasons for such distinct behavior. In this work, the photophysical properties of a functional luminescent system composed of self-assembled Phe-Phe distinct nanostructures combined to polyvinylcarbazole (PVK) are studied. Through Fluorescence Lifetime Imaging Microscopy (FLIM), the intermolecular interactions that are explored in the self-assembling supramolecular strategy to render nanotubes and nanovesicles, as well as to combine PVK, and the unique photophysical properties presented by the resulting materials are scrutinized. FLIM was able to evidence the local fluorescence steady-state and time-resolved behavior, the role of electronic excited states in the efficiency of the energy transfer processes that are enabled by the materials combination and the influence of the intermolecular interactions that result in distinct self-assembled Phe-Phe structures. The results show that these systems present modulated fluorescence emission and enhanced energy transfer efficiency, which turn them into interesting alternatives for application in photoluminescent and photovoltaic devices.

## Introduction

Self-assembled peptides have been widely studied due to the variety of nanostructures that can be obtained, presenting unique functional properties. They can be easily prepared by a variety of supramolecular bottom-up strategies and physically combined to several other materials to have their final properties modulated, which is crucial to find a wide range of application in several technological fields [1–7], including solar cells and sensors [8–10], due to the fact that their unique photophysical properties are closely related to their structural organization. In addition, they undergo natural electron transfer processes that confer them unique photophysical characteristics, including tunable electronic excited states, which is an interesting advantage [11–13]. Moreover, self-assembled peptide nanostructures are efficiently combined to luminescent polymers that interact with them mainly through pi-stacking and electrostatic interactions, which are the more prominent intermolecular interactions driving their self-assembling. In particular, polymers of carbazole derivatives are interesting candidates due to their well-known optical properties and low ionization potential, which enable them to contribute to energy transfer processes when associated with electron acceptors [14]. Among them, poly-(N-vinylcarbazole) (PVK) presents excellent photoconductive properties [15, 16] since it presents a high quantum yield (Φ𝐹 = 0.38) and well-defined spectral profile. The absorption band at 345 nm is due to the excitation of an electron from the ground electronic state to the lowest-energy excited electronic state (𝐿_𝑏_ ⟵ 𝐴_1_) [16]. Is also present a more energetic absorption peak at 295 nm, due to the transition of an electron at the ground state to the second excited electronic state (𝐿_𝑎_ ⟵ 𝐴_1_). Moreover, the fluorescence spectrum presents highly structured vibrational bands, with maxima at 370 and 390 nm.

Due to the ability of carbazole derivatives to form excimers and energy transfer complexes, they present biexponential or multiexponential fluorescence decay curves. In polymeric systems such as PVK, the multiexponential behavior is due to different fluorescence decay lifetimes that are related to three more relevant emitting entities: (1) the longer fluorescence lifetime of around 14 ns that corresponds to the state from where isolated carbazole moieties emit their fluorescence, (2) the shorter lifetime of around 2 ns, which is due to a high-energetic T-shaped excimer and (3) the intermediate lifetime that varies from 5 to 30 ns and is due to a sandwich-like excimer. This behavior of carbazole derivatives, in addition to their photoconductive properties, the use of these materials in optoelectronics has become more popular in recent years. Interesting examples of the variety of application these compounds find are the material synthesized by Nasiri et al. [17] based on carbazole containing tetra-triphenylethylene moieties, aiming to enhance the efficiency of undoped organic fluorescent light-emitting diodes and the system which contains a substituted carbazole as electron donor and CF_3_ units as electron acceptor designed to be applied to ZnO-based dye-sensitized solar cells (DSSC). They also synthesized several carbazole derivatives that found applications as active layer in organic solar cells [18, 19]. From those applications, it is possible to infer that combinations of carbazole derivatives with distinct types of materials is a strategy usually exploited to increase the efficiency of optoelectronic devices, evidencing the great perspectives of such materials in energy conversion applications [20].

However, to understand the nature of such interactions, and predict the photophysical properties that would simplify the processes to propose new materials for energy conversion remain an issue. With this regard, this work presents the FLIM (Fluorescence Lifetime Imaging Microscope measurements) technique as an efficient technique to give more detailed information about the interactions that modulate energy transfer processes, enabling a better understanding of the energetic relations between the excited electronic states of the supramolecular peptide structures and how they are affected by the interaction with carbazolyl groups of the doping polymer. Having this information is crucial to propose a variety of applications, which have been of major interest in the energy conversion field, as it is well demonstrated by the increase of patent applications having self-assembled peptides as subject-matter in several fields of applications. To the date, in a basic search conducted in ESPACENET patent database, there are over 31,000 patent applications focusing on self-assembled peptides to applications such as genetic engineering, medicinal preparations, methods of analyzes, veterinary applications, nanotechnology, adsorption, and electronics, as evidenced by the international patent classification applied to those patent applications.

In this work, we have produced distinct self-assembled Phe-Phe structures doped with PVK and have studied the photophysical behavior of these functional fluorescent systems through steady-state and time-resolved fluorescence spectroscopy, exploring the local area by using FLIM to perform mapping experiments. Results enabled us to infer about the intermolecular interactions that drive the supramolecular self-assembling in the presence of a polymeric doping agent with morphological and photophysical characteristics and infer about the energy transfer processes that confer to the final system unique photophysical properties that can be modulated. The proposed combination in this work presents a promising potential for light-sensitive technological applications.

## Experimental

### Reagents and Equipment

All reagents used in this work were of analytical grade and were used without further purification. Diphenylalanine (Phe-Phe) was purchased from Sigma-Aldrich. Poly(N-vinylcarbazole) (PVK) was obtained from Alfa Aesar. Tetrahydrofuran (THF) and methanol were purchased from Merck. Ultrapure water (Milli-Q system, Millipore) was used for all preparations. Fluorescence measurements were performed in a Horiba FluoroMax 4 spectrophotometer equipped with a 450 W xenon lamp for steady-state measurements. Fluorescence decay curves were recorded in a Horiba DeltaMyc Fluorescence Lifetime Imaging Microscope System (FLIM) that employs time-correlated single-photon counting (TCSPC), objectives of 10-fold, 20-fold and 60-fold magnification, a PPD photodetector, operating in the range of 250–850 nm, with dark counts of less than 80 cps and a CCD camera for wide field imaging. Excitation was promoted by Horiba Direct-coupled pulsed laser DeltaDiodes and DeltaHub of 270, 375 and 450 nm, at a repetition rate of 10 kHz to 100 MHz. According to the excitation source used, a filter in the DeltaMyc microscope is selected to select the emission region to the detected. Thus, with excitation at 375 nm, the filter 3 (cutting 380 nm) of the microscope was used and with excitation at 450 nm, filter4 (500 nm) was selected, preventing the excitation interference on the detected signal. Morphological characterization was carried out using a Jeol JSM7100F (SEM-FEG) with 7 kV electron acceleration voltage in secondary electron detection (SED) mode.

### Phe-Phe Solution Preparation

10 mg Phe-Phe is slowly dissolved in 1 mL 1,1,1,3,3,3-hexafluoropropanol under stirring, rendering a 10 g L-1 stock solution, which is kept under refrigeration and protected from light, in an amber flask.

### Preparation of PVK Solution

A stock solution of PVK was prepared by dissolving 0.1 mg of PVK in 1,1,1,3,3,3-hexafluoropropanol and, then, 10 mL of tetrahydrofuran (THF), resulting a concentration of 10^− 2^ gL^− 1^. This solution was then diluted to a working concentration of 10^− 6^ gL^− 1^ for spectroscopic measurements and sample preparation. The prepared solution was stored in an amber bottle and kept at a temperature of 7.5 °C. Steady-state excitation and fluorescence spectra were obtained for PVK casting films produced from this diluted solution by placing 75 µL over a quartz substrate of 2 cm x 2 cm. Excitation maxima was 324 nm and emission maxima was at 385 nm with a shoulder at 407 nm and another at 362 nm.

### Preparation of Self-Assembled Phe-Phe/PVK Structures

Self-assembled Phe-Phe structures containing the luminescent polymer were prepared in two environments of distinct polarities and polarizabilities, being selected for this the solvents THF and methanol. All self-assembled systems were prepared using a bottom up supramolecular strategy, consisting of dissolving Phe-Phe first in 1,1,1,3,3,3-hexafluoropropanol, then adding a solvent to it and the fluorophore solution. To obtain the system in nonpolar solvent, a 6 µL aliquot of the Phe-Phe stock solution was diluted in THF to obtain a 6 g L^− 1^ Phe-Phe solution that is further diluted to 2 g L^− 1^. To form the self-assembled structures in the solid state, 15 µL of the 2 g L^− 1^ Phe-Phe solution was deposited on a quartz substrate and kept in an oven at 40 °C under vacuum for 48 h for solvent removal.

To prepare the self-assembled system in polar solvent, due to the low miscibility of the solvents, the methodology needed to be adapted. In this procedure, the Phe-Phe stock solution was diluted in methanol to 2 g L^− 1^. Then, 15 µL of this diluted Phe-Phe solution was deposited on a quartz substrate, which was kept in an oven at 40 °C, under vacuum for 48 h. After this time interval, another 15 µL of the PVK solution 10^− 6^ gL^− 1^ in THF were deposited on the already formed structure. After this second deposition, the quartz substrate was kept in an oven at 40 °C for another 48 h.

### Fluorescence Spectroscopy

Steady-state fluorescence excitation and emission spectra of the initial solutions and fluorescence mapping experiment of the solid samples were recorded in a HORIBA Fluoromax-4 spectrophotometer with a 450 W xenon arc lamp, connected to a double monochromator. Mapping regions were selected in the DeltaMyc software, where the local environment of analysis was delimited by a pinhole of 600 μm and the objective of 60-fold magnification.

Time-resolved fluorescence measurements were performed using the time-correlated single photon counting (TCSPC) technique. Fluorescence lifetime single-point and mapping measurements were performed in a DeltaMyc fluorescence lifetime imaging microscope (FLIM) coupled to a HORIBA FluoroMax-4 spectrophotometer. A DeltaDiode excitation source operating at 375 nm, with a pulse width of 20 ps, a DeltaDiode that provides excitation at 450 nm and a DeltaHub controller were used for lifetime acquisition. Fluorescence decay curves were analyzed using a multi-exponential decay model to determine fluorescence lifetimes and their respective contributions. FLIM was used to obtain decay curves at distinct microregions, yielding a distribution of fluorescence lifetimes within the self-assembled structures. The recorded data were analyzed by DAS 6^®^ software embedded in the equipment, which encompasses the known Forster equations for fitting mono, bi and multiexponential decays, FRET distances and efficiencies, requiring simple input, such as fluorescence lifetime of the donor in the absence of the acceptor and the impulse response function (IRF), which is experimentally obtained. Adjustments are evaluated considering the resulting χ^2^ least-square adjustment parameter. Results are, then interpreted, considering their physical meaning.

### Morphological Characterization

Self-assembled Phe-Phe/PVK morphological characterization was performed by a field-emission scanning electron microscope (FE-SEM, Jeol JSM-7100 F) operating at an acceleration voltage of 7 kV in secondary electron detection mode (SED). Samples were prepared by depositing the solutions onto silicon wafers and then sputter-coating with a thin layer of gold to ensure conductivity for SEM imaging.

## Results and Discussion

### Fluorescence of PVK

The photophysical properties of PVK in solution and as thin film were investigated. In THF solution, excitation spectrum showed maximum intensity at 352 nm and fluorescence presented two main peaks at 361 nm and 384 nm (spectra not shown). As reported by Johnson [16], emission maxima near 350 nm are characteristic of the isolated carbazole moiety, suggesting minimal aggregation phenomenon for PVK in THF solution. However, a secondary band extending from 400 nm to 500 nm was still observed, indicating the formation of low- energy aggregates. In this solution, the higher-energy emission band (0,0) presented a decreased intensity due to self-absorption caused by the overlap of excitation and emission spectra. Additionally, the fluorescence observed in the region above 370 nm for this solution is due to intermolecular excimers that are formed when carbazole groups are interacting with each other in a partially overlapped configuration, which originates the T-shaped excimer [21], whereas a fluorescence broad band in the region of 400 nm to 530 nm, is due to the sandwich excimer formation, where a complete overlap of the carbazole unit occurs, leading to greater stability due to increased π-electron cloud overlap.

Time-resolved fluorescence analysis of PVK in THF revealed multiexponential and biexponential behavior within the examined emission range. A lifetime component of 1 to 2 ns was identified, contributing up to 88% to the total decay curve at more energetic emission wavelengths. As the emission wavelength increased, (and energy is lower) a transition to biexponential behavior was observed, characterized by the absence of the shortest lifetime. The longest lifetime, τ_1_, progressively increased with emission wavelength, with values between 18 ns and 22 ns, peaking at 450 nm. Also, at longer wavelengths, the longest lifetime contributes with 83% to the total curve. The τ_2_ component also showed an increase in both its value and contribution to the total curve as emission wavelengths increased. This behavior and the fact that the rise time of the decay curve is shifted, relatively to the instrumental response, an energy transfer process that takes place non-radiatively is expected to occur in the PVK system.

In complex systems, such polymers of supramolecular structures as these studied in this work, fluorescence decay frequently occurs following a multiexponential behavior, due to the fact that these systems present multiple emissive entities and a higher probability of energy transfer through several mechanisms. Therefore, it is crucial to understand the structures and how they influence the electronic organization that promotes the stabilization and destabilization of the excited states, leading to unexpected photophysical properties. The longer lifetimes increase at low-energy wavelengths, and the biexponential behavior of the fluorescence decay suggests that emission is predominantly governed by aggregates present in the solution, as already reported by earlier [22, 23]. In previous studies [23] PVK thin films exhibit a longer fluorescence lifetime of 11 ns, modulated by the presence of aggregates.

### Morphology of Self-Assembled Structures

**Fig. 1:**
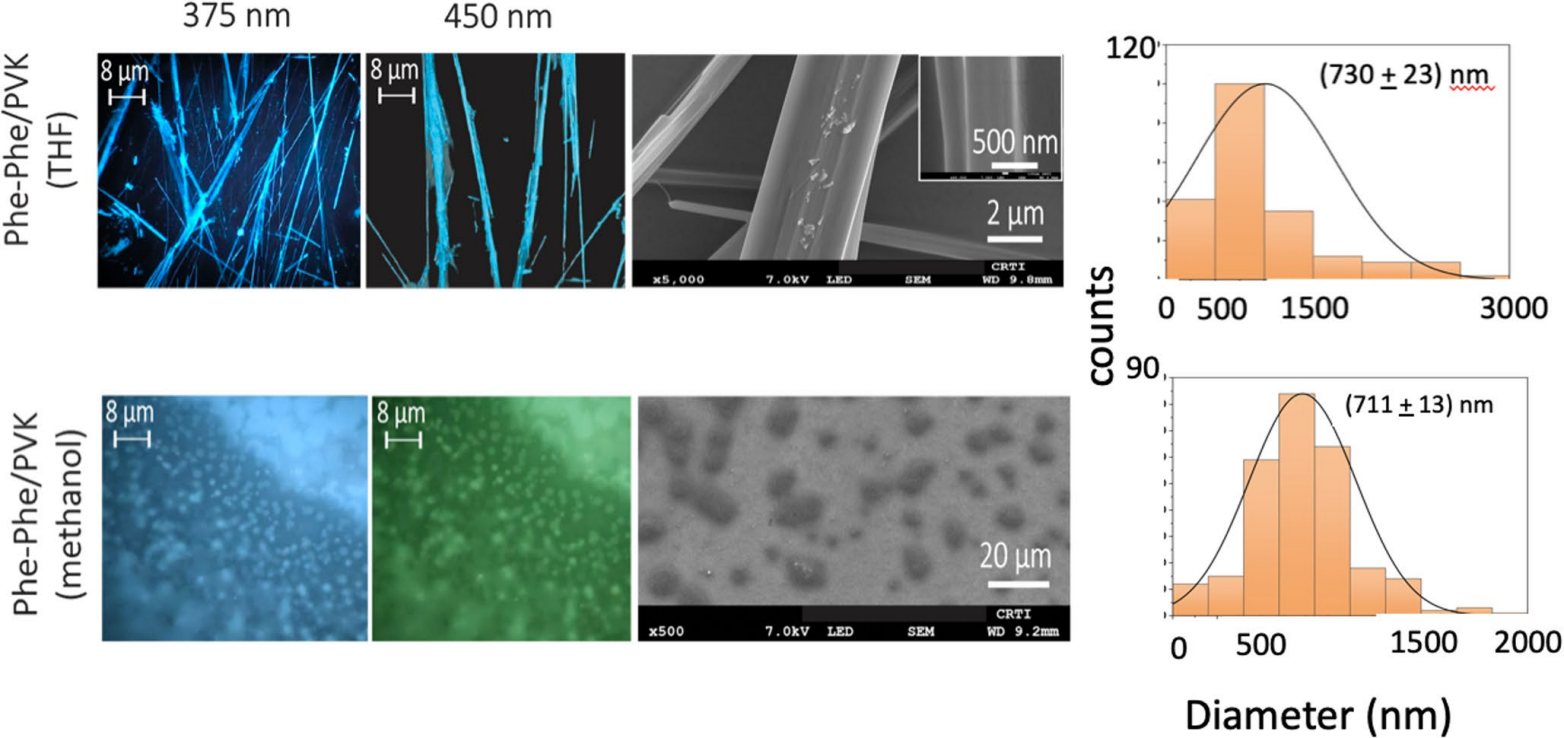
Fluorescence images acquired under excitation of 375 and 450 nm. and scanning electron micrographs and size distribution of the self-assembled Phe-Phe/PVK structures produced in THF and methanol

Morphology of the self-assembled systems was studied by fluorescence imaging and Scanning Electron Microscopy (SEM). Figure 1 shows the fluorescence and SEM images of the self-assembled luminescent Phe-Phe/PVK structures.

In THF, Phe-Phe is known to form nanorod structures. However, when PVK solution is added to the self-assembling environment, there is a change in the hydrophobic/hydrophilic balance of interaction between diphenylalanine monomers and PVK in that solution, leading to a distinct supramolecular self-assembling dynamic, as described elsewhere [24, 25]. Thus, in THF, as shown here, PVK addition to the self-assembling environment influences the process, leading to a preferred nanotube formation. In contrast, Fig. 1 shows that the Phe-Phe self-assembling in methanol and in the presence of PVK results in nanovesicles, due to the polarizability differences of the distinct solvents used to prepare the assembling environment. These structures present emissions in two distinct spectral ranges: around 420 nm, when excitation is performed at 375 nm and 500 nm, with excitation at 450 nm, contrasting with Phe-Phe/PVK nanotubes, which present characteristic emission only in the blue region (at 410 nm). Moreover, the Phe-Phe/PVK system self-assembled in methanol exhibits a background emission that was not observed in THF formed nanotube structures. PVK addition forms a thin film over the previously formed vesicles. The emission of PVK thin films predominantly occurs at 400 nm (blue region of the spectrum) [23], however, when combined with Phe-Phe structures, background emission is red-shifted to around 500 nm, as a result of the interaction between Phe-Phe molecules and PVK moieties at the surface, which results in a stabilization of the electronic excited states of the aggregates.

### Steady-State Fluorescence Studies of Self-Assembled Structures

The steady-state fluorescence studies of the supramolecular structures were carried out in 2 distinct approaches: single-point and mapping experiments.

#### Single-Point

**Fig. 2:**
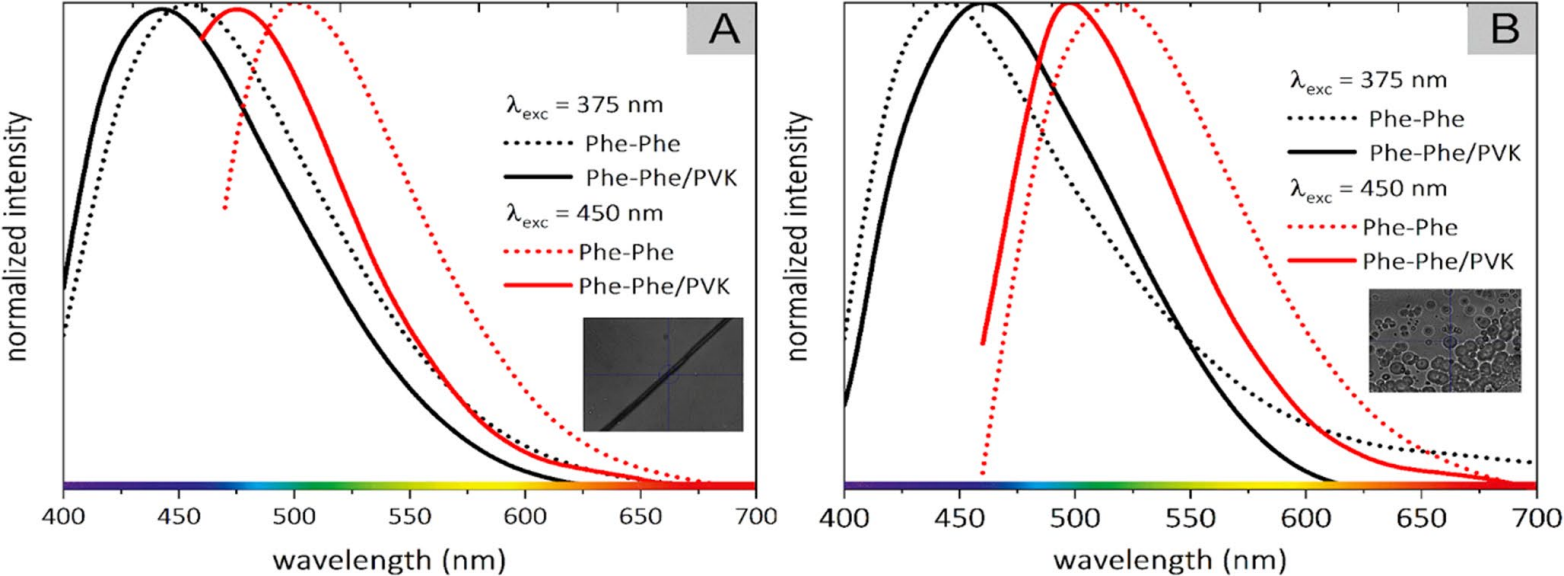
Steady-state fluorescence spectra of Phe-Phe/PVK, self-assembled in (**A**) THF and (**B**) methanol and deposited on a quartz substrate, recorded at distinct excitation wavelengths

Figure 2 presents the fluorescence emission spectra of the Phe-Phe/PVK system formed in THF (Fig. 2A) and methanol (Fig. 2B) and Phe-Phe structures produced in both solvents, but in the absence of PVK, for comparison.

Fluorescence spectra obtained for nanotube structures with excitation at 375 nm and 450 nm, show maxima at 441 nm and 475 nm, respectively, which corroborate with fluorescence observed by fluorescence microscopy (Fig. 1). When compared to the emission of Phe-Phe nanotubes (Fig. 2A), fluorescence of Phe-Phe/PVK nanotube is blue-shifted as excitation is performed both at 375 nm and 450 nm, peaked at 455 nm and 500 nm, respectively. This evidenced that the red-shift is an intrinsic property of Phe-Phe nanostructures, which depends on structural organization, however, when PVK involved in the structure, it interacts with Phe-Phe shifting the emission peaks to shorter wavelengths (442 nm and 475 nm, upon distinct excitation at 375 nm and 450 nm). This spectral blue-shift suggests that the dominant interactions between Phe-Phe and PVK give rise to a distinct electronic excited state, of higher energy and more stable than that of the Phe-Phe nanotube alone. Since PVK is a known semiconductor polymer, it can induce changes in the electronic properties of Phe-Phe nanostructures due to its charge transfer capability [26] and result in distinct photophysical properties of those expected for a Phe-Phe supramolecular structure.

Figure 2B shows the fluorescence spectra of Phe-Phe vesicles and Phe-Phe/PVK vesicles. that Phe-Phe nanovesicles, with excitation performed at 375 nm and 450 nm. Phe-Phe nanovesicles exhibit fluorescence peaks at 444 nm and 517 nm, respectively, which is the intrinsic emission characteristics of Phe-Phe nanovesicles. As for Phe-Phe/PVK vesicles, a fluorescence red-shift is observed, and peaks occur at 459 nm (excitation at 375 nm) and 497 nm (excitation at 450 nm), revealing a dependence of the fluorescence on the final structure of the systems.

**Fig. 3:**
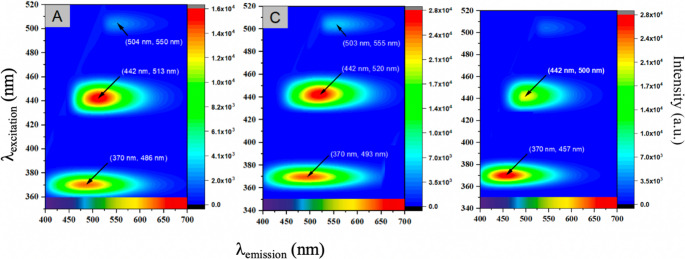
Graphs of the excitation-emission steady-state spectra matrices obtained for (**A**) Phe-Phe/PVK nanotubes; (**B**) Phe-Phe/PVK vesicles and (**C**) Phe-Phe nanotubes produced in water. Excitation range: 360–520 nm. Emission range: 400–700 nm

To understand the fluorescence dependence with the imbalance of interactions that renders distinct supramolecular structures, it is crucial to study the dependence of fluorescence intensity with emission-excitation energies. Therefore, graphs presenting the fluorescence intensity in the Excitation-Emission matrix obtained for Phe-Phe/PVK nanotubes and Phe-Phe/PVK vesicles are presented in Fig. 3. From it, three main emission regions are observed for all systems, resulting from excitation performed at distinct wavelengths. These emissions are also distinct from sample to sample, indicating a dependence with the excitation energy that is also dependent of the structure. For self-assembled Phe-Phe/PVK nanotubes (Fig. 3A), emission ranges from 410 nm to 570 nm when, with a maximum at 486 nm when excitation is performed between 360 nm and 380 nm; fluorescence is observed from 450 nm to 600 nm, with a maximum at 513 nm, when the nanotubes are excited in the range between 430 nm and 450 nm and an emission of low intensity ranging from 530 to 580 nm and peaking at 550 nm, when excitation is performed at the 505–515 nm range. The luminescent behavior of Phe-Phe/PVK vesicles is presented in Fig. 3B, where a slightly distinct behavior is observed for this structure. Fluorescence ranging from 400 to around 630 nm, peaking at 493 nm is obtained upon excitation in the range of 360–380 nm. Also, upon excitation from 430 to 450 nm, fluorescence was red-shifted, occurring in the range from 460 to 620 nm, with a peak at 520 nm and the less intense fluorescence occurring in the range from 540 to 610, upon excitation between 500 and 515 nm, evidencing the red-shift of the fluorescence of vesicles upon energetic excitation, compared to the behavior of the nanotubes, which is indicative of a greater stabilization of the excited states in vesicles than in nanotubes.

Comparing the behavior of the self-assembled structures formed in the presence of PVK with Phe-Phe nanotubes formed in water (Fig. 3C) and in the absence of the polymer, their emission is red-shifted. Phe-Phe nanotubes present blue-shift of the fluorescence bands, regardless the excitation region: a more intense fluorescence is recorded in the region of 390–510 nm when excitation is performed in the range of 360–380 nm, peaking at 457 nm; another band centered at 500 nm with excitation performed at 430–450 nm and a very weak fluorescence centered at 540 nm when excitation is performed at 500–515 nm. This suggests that the interaction with PVK leads to a general stabilization of excited states, enabling fluorescence modulation by combination with the polymer.

#### Steady-State Mapping

**Fig. 4:**
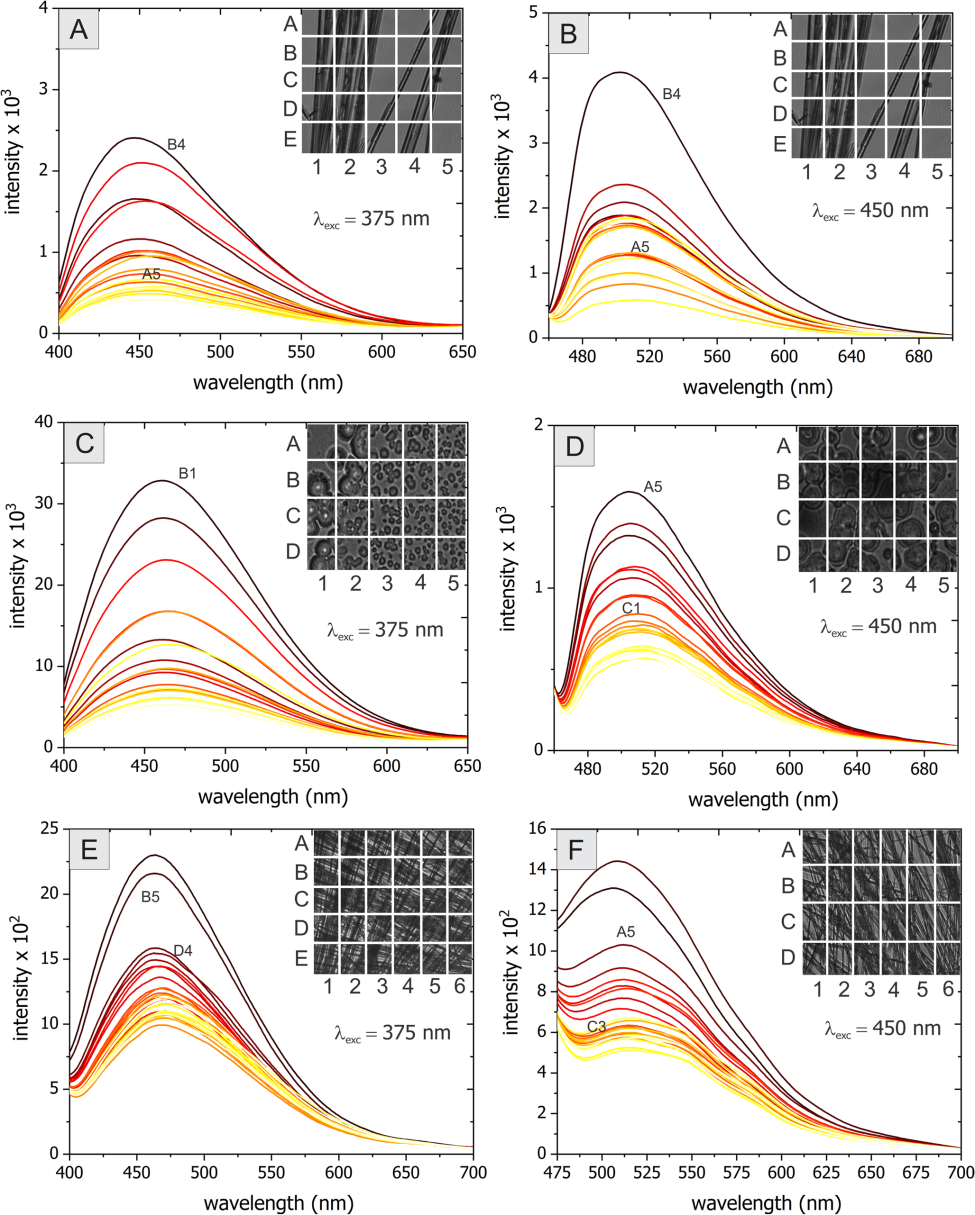
Fluorescence emission spectra mapping of self-assembled Phe-Phe/PVK nanotubes (**A**) upon excitation at 375 nm; (**B**) upon excitation at 450 nm; Phe-Phe/PVK nanovesicle (**C**) upon excitation at 375 nm and (**D**) upon excitation at 450 nm; and Phe-Phe nanotubes prepared in water (**E**) upon excitation at 375 nm; (**F**) upon excitation at 450 nm

To understand the local contribution to the total fluorescence emission of each system, steady-state fluorescence mappings of Phe-Phe/PVK systems were performed, by obtaining fluorescence spectra of local environment, selected by the Delta Myc objective. For all measurements, a pinhole of 600 μm and an objective of 60-fold magnification were used. Upon a chosen excitation, steady-state fluorescence spectra were recorded at each region that constitutes the total mapped region. Mappings of Phe-Phe/PVK nanotubes and nanovesicles are shown in Fig. 4.

From Fig. 4, regardless of the excitation wavelength, regions with few or isolated structures (nanotube or nanovesicles, depending on the sample) show higher fluorescence intensity compared to regions with higher structure density, as evidenced by comparing the fluorescence intensity of marked spectra, for instance, in Fig. 4A, which shows the mapping recorded for Phe-Phe/PVK nanotubes with excitation at 375 nm, spectrum of the region A5 is less intense than spectrum of region B4. Also in Fig. 4C, which shows the mapping recorded for Phe-Phe/PVK nanovesicles with excitation at 375 nm, spectrum of B1 region is the most intense. This evidence that these structures are at an adequate distance for energy transfer, leading to the deactivation of the excited states of the donor and less intense resultant fluorescence. In addition, when compared to the mapping of Phe-Phe nanotubes self-assembled in the absence of PVK (Fig. 4E and F), spectra are at least 10 times less intense than spectra recorded for the structures formed in the presence of PVK, which may be due to the fact that there is a significant spectral overlap between the emission spectrum of PVK and the excitation spectrum of Phe-Phe structures. Also, upon less energetic excitation (450 nnm), the emission spectra present a composition of overlapped peaks that are not shown in the structures formed in the presence of PVK, indicating that there must be a participation of distinct nanotubes in the fluorescence spectrum of each microenvironment. Therefore, the presence of PVK significantly increases the overall fluorescence intensity of the system. Furthermore, the presence of PVK results in a much more significant loss of intensity in regions with a higher presence of self-assembled structures than that observed in the mapping of Phe-Phe nanotube in the absence of PVK. This may indicate that PVK, by interacting with Phe-Phe, forms a supramolecular system that facilitates energy transfer more efficiently, which can be evidenced by time-resolved measurements.

Figure 4 also evidences the excitation dependence of the steady-state fluorescence spectra intensity of the systems studied here, From Fig. 4A and B, fluorescence mapping of Phe-Phe/PVK nanotubes presents overall more intense spectra upon less energetic excitation (450 nm) than those recorded with excitation at 375 nm. Also, spectra are red-shifted upon less energetic, presenting fluorescence maxima at around 510 nm, whereas, upon excitation at 375 nm, the fluorescence maxima are centered at 460 nm. This same red shift is observed for nanovesicles (Figs. 4C, D); however, spectral intensity is 20-fold higher when excitation is at 375 nm, evidencing that the non-radiative energy transfer is more efficient in nanovesicles than in nanotubes. For Phe-Phe/PVK vesicles, similarly to what was observed for nanotubes, the fluorescence spectra are also red-shifted, presenting a maximum of around 510 nm upon less energetic excitation and 460 nm with excitation performed at 375 nm. To better understand the nature of this behavior, time-resolved measurements are needed.

### Time-Dependent Fluorescence Measurements

Time-resolved fluorescence spectroscopic studies of the supramolecular structures were carried out in 2 distinct approaches: single-point and mapping experiments, using the TCSPC technique.

#### Time-Resolved Single-Point Fluorescence Measurements

In the single-point experiment, an exploratory measurement was carried out to obtain information about the overall deactivation behavior of the self-assembled Phe-Phe/PVK systems. Therefore, fluorescence decay curves were recorded, and they showed that Phe-Phe/PVK nanotubes and nanovesicles exhibit multiexponential behavior, which is expected for the heterogeneous supramolecular structures, such as those formed in these systems. The fluorescence lifetimes obtained for Phe-Phe and Phe-Phe/PVK nanotubes and nanovesicles are presented in Table 1.

**Table 1:** Fluorescence lifetimes obtained for PVK film, Phe-Phe nanotubes and Phe-Phe/PVK nanotubes and nanovesicles, with excitation at 375 and 450 nm

$$\lambda_{exc}=375 nm$$			
	${\tau}_1(ns)$	${\tau}_1(ns)$	${\tau}_3(ns)$
Phe-Phe (nanotube)	0.228 (64%)	1.593(29%)	5.435(7%)
Phe-Phe (vesicle)	0.206(78%)	1.289(19%)	4.916 (3%)
Phe-Phe/PVK (nanotube)	0.304(61%)	1.702(32%)	5.734(7%)
Phe-Phe/PVK (vesicle)	0.378(47%)	1.776(39%)	5.194(14%)
PVK	0.530(79%)	2.334(19%)	8.382(2%)

$$\lambda_{exc}=450 nm$$			
Phe-Phe (nanotube)	0.186(73%)	1.119(22%)	4.881(5%)
Phe-Phe (vesicle)	0.295(70%)	1.674(25%)	5.151(5%)
Phe-Phe/PVK (nanotube)	0.247(54%)	1.378(31%)	4.499(15%)
Phe-Phe/PVK (vesicle)	0.459(44%)	2.025(39%)	5.318(17%)
PVK	0.618(84%)	2.278(14%)	7.263(2%)

From the data presented in Table 1, it is noteworthy that Phe-Phe/PVK nanotubes present longer lifetimes than nanovesicles when excitation is performed at shorter wavelengths. When excitation is performed at 375 nm, Phe-Phe/PVK nanotubes present a longer lifetime of 5.7 ns and contributing with 7% of the total decay curve; an intermediary lifetime of 1.7 ns (with contribution of 32%) and a shorter lifetime of 0.3 ns (and contributing with 61% of the total decay curve), while Phe-Phe/PVK vesicles present a longer lifetime of 4.5 ns, but with greater contribution to the total decay curve (14%); an intermediary lifetime of 1.3 ns (32%) and a shorter lifetime of 0.25 ns (47% of contribution). When excitation is performed at a longer wavelength (450 nm), nanovesicles present longer lifetimes than those of nanotubes, being 5.3 ns and contributing with 17% of the total decay curve; the intermediary life-time being 2.0 ns (39%) and the shorter lifetime being 0.45 (44%), while nanotubes present the longer lifetime of 5.1 ns (14%); an intermediary of 1.8 ns (39%) and a shorter lifetime of 0.38 ns (47%). These data evidence that, upon distinct excitation energies, excited electronic states of the supramolecular structures become more stable; however, to deepen our understanding of the lifetime behavior of self-assembled Phe-Phe/PVK systems, a comparative analysis of the lifetimes of these structures in the absence and presence of PVK was conducted.

In Table 1, lifetimes of the Phe-Phe/PVK systems are intermediate between those of shorter lifetimes of the isolated Phe-Phe structures and the longer lifetimes of PVK films. This behavior demonstrates that the supramolecular structures formed in the presence of the polymer exhibit peculiar photophysical characteristics, clearly distinct from those of the isolated components. Although steady-state fluorescence analysis, discussed in Sect.  3.3, had already evidenced that the system presents these distinct photophysical properties, lifetime analysis confirms the existence of electronic states intrinsic to the Phe-Phe/PVK system, strongly related to the peculiar interactions of PVK with Phe-Phe structures. Whereas PVK is a semiconductor polymer presenting unique electronic and optical properties, including a relatively long lifetime fluorescence due to stable electronic excited states in this polymer, Phe-Phe nanostructures present shorter fluorescence lifetimes due to their molecular structure that favors faster relaxation processes. When PVK is combined with Phe-Phe nanostructures, their π-π interaction favors energy transfer processes between the two components that are not possible in isolated PVK, resulting in the instability of the excited states in the combined system compared to the isolated PVK. Due to this effect, more efficient non-radiative relaxation mechanisms are possible in the Phe-Phe/PVK nanostructures. On the other hand, it is clear that PVK can stabilize the electronic excited states of Phe-Phe nanostructures by preventing some of their natural relaxation processes. This stabilization leads to the observed longer fluorescence lifetimes in the Phe-Phe/PVK systems than in isolated Phe-Phe nanostructures, due to a synergistic interaction between PVK and Phe-Phe nanostructures that creates a dynamic equilibrium in the electronic states, providing a material with flexible and modulating photophysical properties. However, it is crucial to fully understand the deactivation mechanisms of the excited states of these systems, which can be achieved by performing FLIM mapping experiments.

#### Fluorescence Lifetime Mapping and FRET

**Fig. 5:**
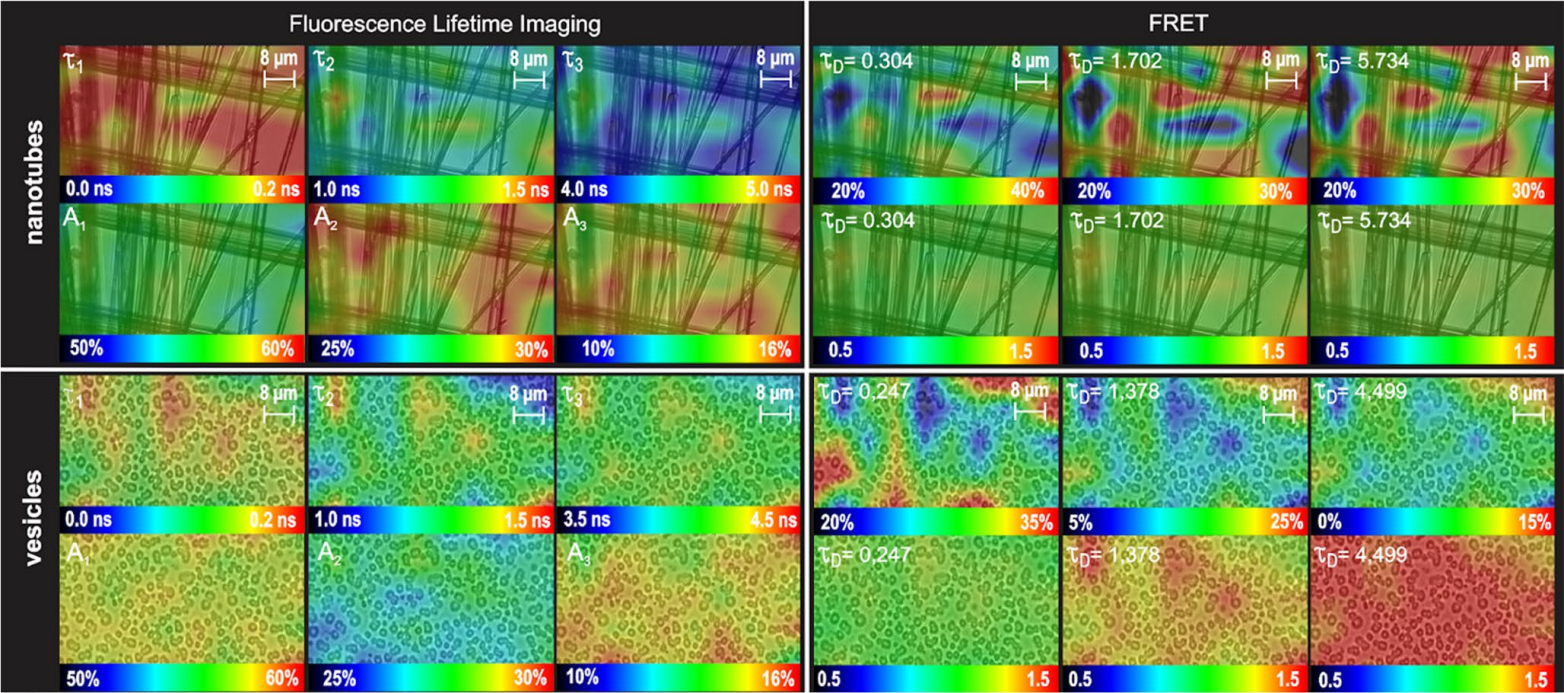
Fluorescence lifetime mapping results for Phe-Phe/PVK samples produced in THF (nanotubes) and methanol (vesicles) with excitation in 375 nm. Column 1: local fluorescence lifetimes and contributions (pre-exponential factor A); column 2: FRET efficiency (%) and Forster distances

The results obtained from the mapping are presented in Fig. 5 and they provide information on the dynamics of the electronic excited states of the self-assembled structures. Results presented in Fig. 5 refer to mapping experiment conducted with excitation at 375 nm.

It is noteworthy that, in nanotubes, lifetime heterogeneity is more pronounced compared to nanovesicles, which is due to the heterogeneity of the sizes and shapes of the nanotube structures that confer a multiexponential behavior for the fluorescence lifetime. For these nanotubes, τ_1_ is close to 0.2 ns, with contributions reaching up to 60%. Its τ_2_ is around 1.2 ns, with contribution of 30% at most and τ_3_, the longer lifetime, is around 4.2 ns, with contributions of 12%.

**Fig. 6:**
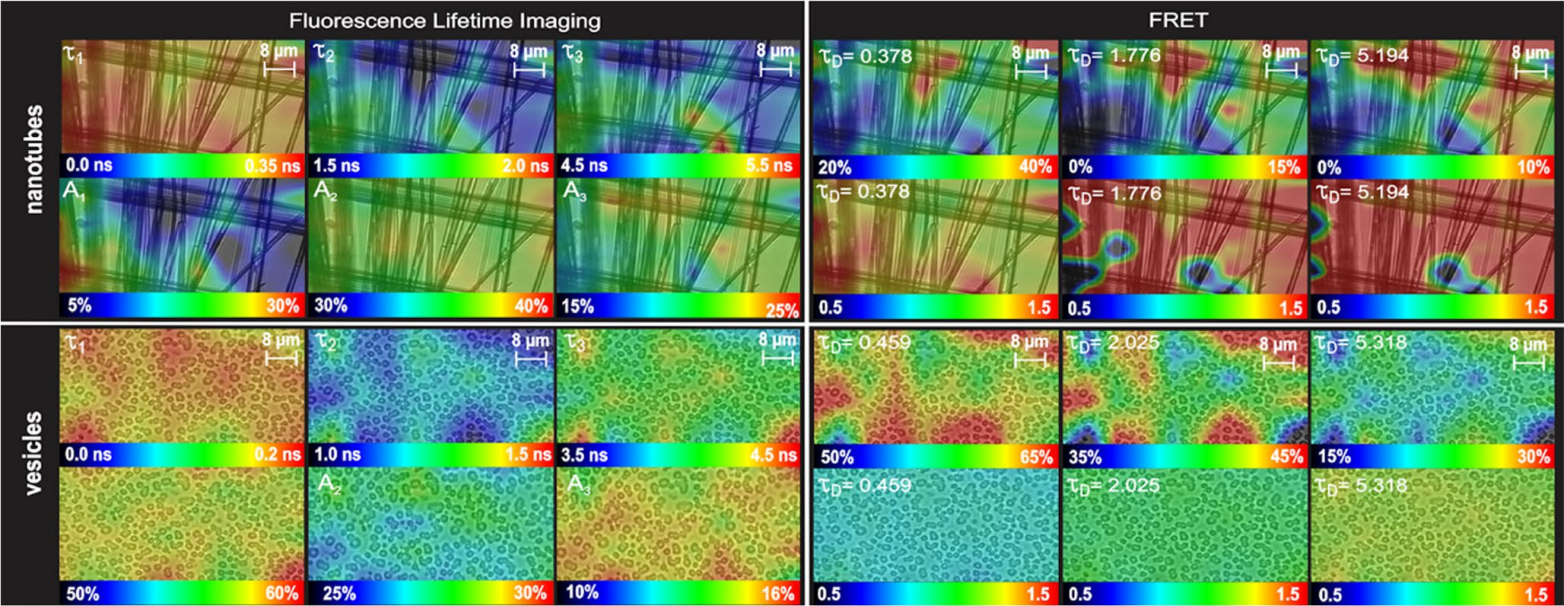
Fluorescence lifetime mapping results for Phe-Phe/PVK samples produced in THF (nanotubes) and methanol (vesicles) with excitation in 450 nm. Column 1: local fluorescence lifetimes and contributions (pre-exponential factor A); column 2: FRET efficiency (%) and Forster distances

Fluorescence lifetimes in nanovesicles tend to be more uniform, as are the contributions, although fluorescence lifetimes of vesicle systems are similar to those of nanotubes, with τ_1_ being around 0.15 ns, with contributions of 50%; τ_2_ of around 1.3 ns, with contribution of 27% and τ_3_ of around 4.0 ns, with contributions also of 12%. Although lifetimes are similar in both types of structures, FRET in nanotubes is more efficient than in vesicles. Figure 5 provide information on the microregions where FRET is more probable, evidencing the heterogeneity of the process in nanotubes, but also evidencing that the process is occurring in the range of Forster distance between donor and acceptor moieties in the system, with efficiency that reaches 30%, while in nanovesicles, the donor is short-lived and the distances between donor and acceptor are virtually higher than the optimal Forster distance, providing that the main mechanism for energy transfer in the vesicle systems is the trivial one. In those systems, FRET efficiency is not superior to 20%, although it is efficient at short-lived states, where Forster distance is optimal and efficiency is, then around 30%. However, it is important to understand if excitation energy can modulate the energy transfer dynamic in such systems. Therefore, mapping was also performed with excitation at 450 nm and results are presented in Fig. 6.

Upon less energetic excitation, the photophysical behavior of both types of structures is completely different. The overall lifetimes obtained for nanotubes are longer than those observed with excitation at higher energy, whereas the lifetimes of the vesicle systems seem to be similar to those registered with excitation of higher energy. For the nanotube systems, the shorter lifetimes τ_1_ is of around 0.35 ns with a small contribution of around 7%. The intermediate lifetime, τ_2_, is 1.6 ns, with 27% of contribution to the total decay curve τ_3_ is of 4.0 ns, with contribution of 15%. For the nanovesicles the shorter lifetimes τ_1_ is of very short, being 0.15 ns and contributing with 55% to the total decay curve; τ_2_, is 1.0 ns, contributing with 35% to the total decay curve and the longer lifetime, τ_3_, is of 4.7 ns, with contribution of 20%. Also, with excitation performed at lower energies, FRET efficiency is higher in vesicles than in nanotubes, again, due to the fact that the excited states reached upon excitation are located in regions that do not comply with the requirement of the optimal distance relative to the acceptor. In nanotubes, Forster distances (r/R0) are close to 1.5, therefore, excited states are long-lived and more suitable for trivial energy transfer mechanism. In contrast, vesicle systems present the Forster distance near 1, in a homogeneous distribution across almost the entire region mapped, resulting in an overall FRET ranging between 30% and 40%. Based on data in Table 1 regarding fluorescence lifetimes of the structures formed only with Phe-Phe, it is clear that addition of PVK to self-assembled Phe-Phe structures improves the homogeneity of Förster resonance energy transfer in these structures, compared to FRET that occurs only in Phe-Phe structures, however, the efficiency can be further improved by controlling excitation energy, to reach the electronic states that will be suitable for FRET, adding the ability to also modulate the balance of energy transfer mechanism, which can be an advantage aiming a technological application. Since self-assembly of Phe-Phe can lead to a variety of structural configurations that cannot always be uniform, leading to variations in the distance and orientation between donor and acceptor molecules, this can also lead to less homogeneous FRET. When PVK is added to Phe-Phe nanostructures, it may interact with the peptide structures to provide more uniform and stable configurations or, at least, to provide a way to manipulate the balance between the possible energy transfer processes that are occurring in such heterogeneous systems. PVK is, in fact, enabling a more consistent donor and acceptor moieties alignment. Additionally, the electronic properties of PVK is also interacting with Phe-Phe structures stabilizing their electronic excited states, further contributing to FRET homogeneity.

## Conclusion

Photophysical properties of Phe-Phe/PVK systems and the isolated materials are distinct, which is due to the nature of interaction between Phe-Phe and PVK that leads to distinct electronic states, characteristic of the multiple component structures, which lead cto distinct photophysical properties, as proved by steady-state and time-resolved fluorescence studies provided, in this work, by FLIM experiments. Here, two main structures were selectively produced and studies: nanotubes and nanovesicles, both combined with PVK. The systems present a red-shifted fluorescence, comparing to that of the Phe-Phe structures, without PVK. However, nanovesicles present an even more red-shifted spectra, occurring in regions that vary with excitation, evidencing its dependence with excitation energy. In addition, the higher fluorescence intensity of the combined system compared to isolated Phe-Phe suggests that a more efficient energy transfer is occurring in the systems.

Fluorescence lifetimes of the Phe-Phe/PVK systems present multiexponential fluorescence lifetime behavior, evidencing the complexity of these systems and corroborating the occurrence of energy transfer processes that are competing with each other. By mapping experiments, FRET was proved to occur in all systems, but differently. In general, FRET efficiency in the Phe-Phe/PVK system surpasses that observed in isolated Phe-Phe structures, demonstrating that the synergistic effect of the combination of these materials can be explored for applications requiring efficient energy transfer. However, the dependence of the energy transfer processes on the excitation and the structural heterogeneity was evidenced, which enabled an energy transfer modulation that can be of crucial relevance in finding a technological application for such materials. The use of FLIM to map the local photophysical behavior of such systems was also crucial to reveal the significance of molecular interactions in nanostructures to give rise to systems with unique and enhanced photophysical properties and to enable a confident relation between structure, preparation details and excitation energy to provide a way to predict and even to control the photophysical properties of complex systems, which is, nowadays, a desirable feature of new materials, especially for energy conversion. These findings open a perspective to use FLIM also to study give information on the electronic excited states of materials and the most probable energy transfer processes, which is crucial information for technological applications.

## Data Availability

Data presented in this work is available in MARTINS, TATIANA (2026), “FLIM Phe-Phe PVK”, Mendeley Data, V1, doi: 10.17632/kmvt3558m7.
